# Investigation of compatibility of severe acute respiratory syndrome coronavirus 2 reverse transcriptase-PCR kits containing different gene targets during coronavirus disease 2019 pandemic

**DOI:** 10.2217/fvl-2020-0169

**Published:** 2020-08-26

**Authors:** Figen Sarıgül, Osman Doluca, Sıla Akhan, Murat Sayan

**Affiliations:** 1 Health Sciences University, Antalya Education & Research Hospital, Infectious Disease & Clinical Microbiology, Antalya, Turkey; 2 Izmir University of Economics, Department of Biomedical Engineering, Izmir, Turkey; 3 Kocaeli University, Faculty of Medicine, Infectious Disease & Clinical Microbiology, Kocaeli, Turkey; 4 Kocaeli University, Faculty of Medicine, Clinical Laboratory, PCR Unit, Kocaeli, Turkey; 5 Near East University, DESAM Institute, Nicosia, Northern Cyprus

**Keywords:** coronavirus, COVID-19, RT-PCR, SARS-CoV-2

## Abstract

**Aim:**

In the diagnosis of severe acute respiratory syndrome coronavirus 2 (SARS-CoV-2), reverse transcriptase-PCR (RT-PCR) technique is often used. We evaluated the compatibility of SARS-CoV-2 RT-PCR kits containing different gene targets during the pandemic.

**Materials & methods:**

Samples were tested by Bio-Speddy^®^ (*RdRp* gene) and Diagnovital^®^ (*RdRp + E* genes). The correlation between two assays were determined by Deming regression analysis and chi-square analyses.

**Results:**

Diagnovital PCR kit showed amplification in a narrow Ct range and conveniently sharper exponential amplification curves than Bio-Speedy PCR kit. While the correlation between the findings of the two kits was apparent even with single gene target, this correlation increased when a secondary biomarker was added to the correlation calculations.

**Conclusion:**

We have observed high correlation between different PCR kits, however, using different PCR kits during the pandemic may provide a more accurate diagnosis of SARS-CoV-2, since despite correlation there are a number of patients showing contradicting diagnosis.

The disease-causing factor was determined to be a new coronavirus after reports of pneumonia cases identified in Wuhan Province of China in December 2019 to the WHO regional office. It was declared as a pandemic by WHO on 11 March 2020. The new coronavirus was named severe acute respiratory syndrome coronavirus 2 (SARS-CoV-2) and has a much higher transmission rate than known human coronavirus strains, while also causing damage to the lung tissue, causing respiratory failure and potentially leading to death [1]. At the beginning of July 2020, the number of SARS-CoV-2 positive cases reported worldwide exceeded 11 million with deaths of 528,204 [2,3]. In Turkey, the first cases were detected on 11 March 2020 and 206,844 cases and 5241 death were present at the beginning of July 2020 [4]. According to new phylogenetic analysis of the virus in Turkey; the introduction of the virus into the country for the first time is earlier than the first reported case of infection, and the virus was found to have many independent international entries into the country [5].

First of all, to control coronavirus disease 2019 (COVID-19) pandemic, early detection and isolation of asymptomatic and symptomatic infections are crucial. Diagnostic tests for SARS-CoV-2 are still developing and a clear understanding of the nature of the tests and interpretation of their findings is essential. For the diagnosis of SARS-CoV-2, reverse transcriptase-PCR (RT-PCR) and IgM and IgG ELISA tests are often used [6,7]. It remains unclear whether RT-PCR is the gold standard and whether false positive or false negative results are common. CDC of United States recommend the collection of oropharyngeal and nasopharyngeal swab specimens to test for SARS-CoV-2 [8]. Various RNA gene targets are used by different manufacturers; most tests target envelopes (*E*), spike (*S*), nucleocapsid (*N*), RNA-linked RNA polymerase (*RdRp*) and *ORF1* genes 1 or more [9]. Among these assays, the *RdRp* assay had the highest analytical sensitivity (3.8 RNA copies/ml reaction at 95% detection probability) [10]. In Turkey, the diagnosis was made according to the recommendations given by ‘COVID-19 Diagnosis and Treatment Guide’ published by the Turkish Ministry of Health [11].

No international external quality controls for the PCR kits used have been established yet. Therefore, multiple diagnostic tools are preferred for correct diagnosis. Here we evaluated the compatibility of two different RT-PCR methodologies used in the simultaneous detection of two regions of the SARS-CoV-2 genome (*E* and *RdRp* genes) and the *RdRp* gene. We have compared the correlation between the results though regression and chi-square analysis and evaluated their effectiveness for the diagnosis.

## Materials & methods

A total of 96 patients’ oro/nasopharyngeal swab samples from different hospitals in Kocaeli city (Kocaeli State Hospital, Seka State Hospital, Gebze Darıca State Hospital and Gölcük State Hospital) which were sent for SARS-CoV-2 diagnosis to PCR unit of Kocaeli University were included to this study between March and April 2020. All samples were studied by a comparison of two different RT-PCR kits produced in Turkey.

One of the RT-PCR kits, the Bio-Speddy^®^ (Bioeksen R&D Technologies Inc. COVID-19 RT-qPCR Detection Kit v2.0, Istanbul, Turkey), is determined valuable by the ‘Turkish Ministry of Health’ and used throughout COVID-19 pandemic. The alternative kit; Diagnovital^®^ (RTA Laboratories Inc, SARS-CoV-2 Real-Time PCR Kit v2.0, Istanbul, Turkey) is a commercial kit and mainly exported to European and Middle East-North Africa zones during the pandemic. Each RT-PCR kit production followed CDC’s and WHO’s detection guidelines [12,13]. However, each kit is included in the WHO Emergency Use Listing for SARS-CoV-2 *in vitro* diagnostic products [14] However, Diagnovital kit has also received ‘The Emergency Use Authorization’ by the US FDA [15]. The Emergency Use Authorization allows the FDA to help strengthen the nation’s public health protections against chemical, biological, radiological and nuclear threats by facilitating the availability and use of the medical countermeasures needed during public health emergencies. Diagnovital kit has been categorized by FDA on the ‘H’ level: laboratories certified under the Clinical Laboratory Improvement Amendments of 1988, 42 U.S.C. §263a, that meet requirements to perform high complexity tests.

Viral RNA extraction from samples were performed according to the manufacturer’s instructions. For automated viral nucleic acid extraction processing Qiagen – EZ 1 Advanced XL platform (Qiagen, Hilden, Germany) and for PCR assay run Qiagen – Rotorgene Q thermal cycler platform (Qiagen) was used. A negative (human specimen control) was included in every RNA extraction procedure, and a nontemplate (water) control was included in every RT-PCR run. An internal control amplification was performed to monitor RNA extraction and RT-PCR quality. Characteristics of the SARS-CoV-2 RT-PCR kits are shown in Table 1.

**Table 1.** Comparison of characteristics the severe acute respiratory syndrome coronavirus 2 PCR kits which analyzed in the study.

Characteristic	SARS-CoV-2 RT-PCR kit	
Brand	Bio-Speddy^®^	Diagnovital^®^
PCR assay	Real time, single-step RT-PCR	Real time, single-step RT-PCR
Amplification assay	Qualitative	Qualitative
Testing specimen	Naso/oropharyngeal aspirates, washes and swabs, bronchoalveolar lavage, tracheal aspirates and sputum	Naso/oropharyngeal aspirates, washes and swabs, bronchoalveolar lavage, tracheal aspirates and sputum
Sample collection	Viral transport medium and swab are component of kit	Viral transport medium must be provided, swab is component of kit
Viral nucleic acid extraction	Manual and component of the kit	Manual or automated and must be provided
Extraction processing time (min)	10	10–45
PCR assay run time (min)	101	119
SARS-CoV-2 gene target	*RdRp*	*RdRp + E*
Internal control target	*RNAase P* gene	*RNAase P* gene
Positive control	Noninfectious	Noninfectious
Storage of the kit	-20°C	-20°C
Analytical sensitivity claim	5.6 copies/ml	10 copies/ml
Result interpretation	*RdRp* detection, amplification must be <40 Ct	*RdRp + E* detection, amplification must be <35 Ct

Ct: Cycle threshold; *E*: Envelop; *RdRp*: RNA-dependent RNA polymerase; RT-PCR: Reverse transcription-PCR; SARS-CoV-2: Severe acute respiratory syndrome coronavirus 2.

Swab materials available for sample collection for COVID-19 based on the CDC Interim Guidelines for Collecting, Handling and Testing Clinical Specimens from Patients Under Investigation for COVID-19 [16]. Oropharyngeal/nasopharyngeal samples were collected from the patients by synthetic fiber swabs with plastic shafts (Citotest Scientific Co, Haimen City, PR China). The swabs were placed in 3 ml sterile viral transport media (Citotest Scientific Co) during the collection and transferred with biohazard specimen bag. After the samples were taken, they were transferred to the center and tested shortly within the 2 h. Samples were vortexed for 3–5 s prior to testing and a calibrated pipette was used to transfer the sample volume specified in each manufacturer’s instructions for use.

### Ethical approval

This study was approved by the COVID-19 Scientific Research Committee of Ministry of Health (S.B. 2020-05-13T14_11_33) and by local Non-Interventional Research Ethics Committee of Kocaeli University (GOKAEK-2020/08.30 – 2020/144). Informed consent was not obtained.

### Statistical analysis

The chi-square tests were calculated using a significance level of 95%. Chi-square scores were calculated using Excel. The heatmap is obtained by comparing chi-square scores for a range of threshold cycle combinations, where any patient with a Ct value below the threshold is assumed positive for Bio-speedy and any patient with a Ct value below the threshold for both of the genes (gene E and RdRp) is assumed positive for Diagnovital.

For Deming regressions, the data pairs with only positive Ct values were used for each experiment, except cumulatives. To obtain the cumulative Ct scores, an arbitrary copy number was obtained using 2^-Ct^ for gene E and RdRp obtained by Diagnovital, where the Ct is available. Otherwise, an arbitrary copy number of zero is used. The arbitrary copy numbers were added to obtain a cumulative copy number, and the cumulative Ct is obtained by ln (cumulative copy number)/ln(2). Calculations were done using Excel.

## Results

Of all analyzed samples, 68 were positive and 28 were negative by two PCR kits. The mean age of the study population was 44 (range: 17 to 85) and 67 (64%) were male, including both genders showing signs and/or symptoms of COVID-19 infection. Demographic, laboratory and medical findings of the study patients are shown in Table 2.

**Table 2.** Demographic, laboratory and medical findings of the study patients.

Characteristic	Patient group
Patient, n	96
Gender, M/F, n (%)	67 (64)/32 (33)
Age, years mean, range	44 (17–85)
Sampling, region/city	Marmara/Kocaeli
**Clinical status, n (%)** **Symptomatic patients** – Cough – Fever + cough + headache + sore throat – Cough + respiratory distress – Fever – Sore throat + cough – Fever + cough – Cough + fatigue + headache – Fever + fatigue + myalgia – Fever + cough + malaise – Malaise + sore throat + myalgia – Cough + fatigue + sore throat – Fever + cough + loss of taste – Fever + abdominal pain + vomiting **Asymptomatic patients**	**62 (65)** 15 (24) 9 (14) 7 (11) 6 (10) 6 (10) 5 (8) 3 (5) 3 (5) 2 (3) 2 (3) 2 (3) 1 (2) 1 (2) **34 (35)**
**Working status, n** (%) – Worker – Self-employed – Housewife – Public personnel – Retired – Health employee – Unemployed	**50 (52)** 20 (40) 8 (16) 6 (12) 6 (12) 5 (10) 4 (8) 1 (2
**Cormorbidity, n (%)** – Hypertension – Diabetes mellitus – Diabetes mellitus + hypertension – Chronic obstructive lung diseases – Cardiac diseases	**12 (13)** 4 (33) 3 (25) 2 (17) 2 (17) 1 (8)
**Computerized tomography status, n** (%) – CT positive patient – CT negative patient – CT not done	29 (30) 37 (39) 30 (31)

CT: Computerized tomography; F: Female; M: Male.

**Figure 1.**
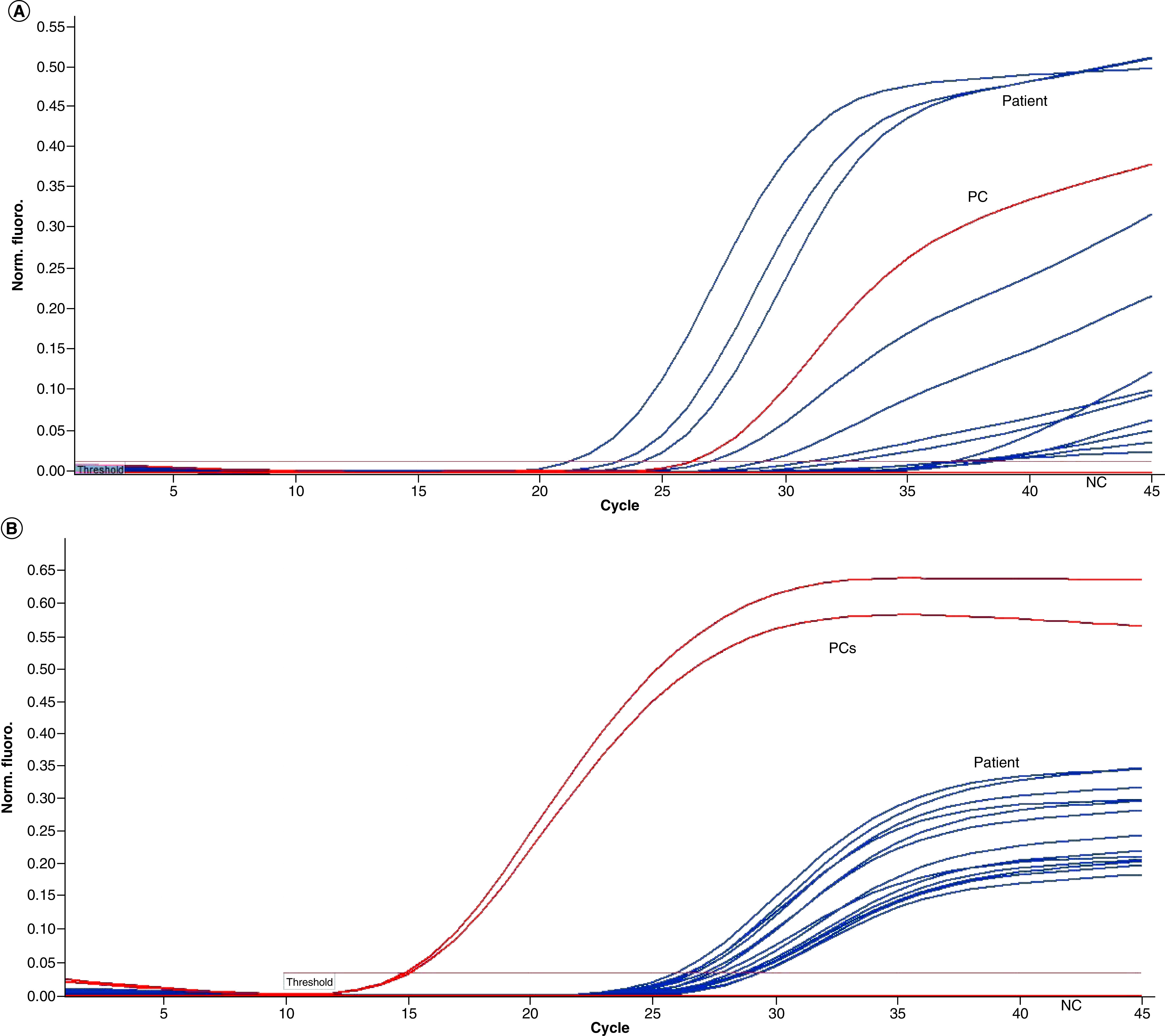
**Reverse transcriptase-PCR amplification yield results.** BioSpeedy^®^ SARS-CoV-2 PCR (*RdRp*) kit **(A)**, Diagnovital^®^ SARS-CoV-2 PCR (*RdRp*) kit **(B)** in Rotor-Gene Q Software 2.3.1. NC: Negative control; PC: Positive control; SARS-CoV-2: Severe acute respiratory syndrome coronavirus 2.

Diagnovital and Bio-speedy kits were compared by RT-PCR amplification results. The initial quantity and yield quality of targets had been monitored by the exponential amplification phase of the RT-PCR reaction. There were different Cts for the same patient samples and each kit indicated differently yield quality. Especially, Diagnovital showed in a constricted range and conveniently exponential amplification curves than Bio-speedy (Figure 1).

**Figure 2.**
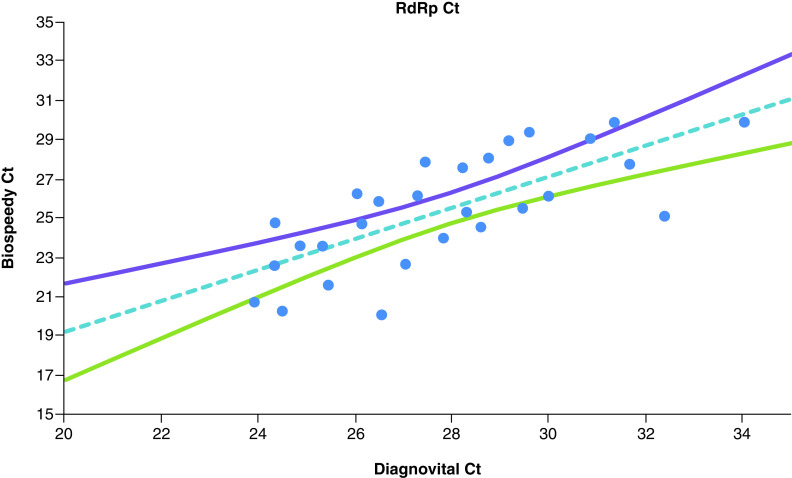
The Deming regression of Ct values obtained for *RdRp* gene for the two separate kits and 95% CI for the regression line. Any data pair with any negative results are omitted.

The Deming regression of Ct values obtained for *RdRp* gene for the two separate kits and 95% CI for the regression line shown in Figure 2. Diagnovital and Bio-speedy both use *RdRp* as a biomarker for their detection. The regression of Cts obtained for this biomarker showed positive correlation. Bio-speedy showed higher variation when it comes to Cts, yet the correlation was statistically significant (p < 0.01) with a Pearson correlation of 0.73 (Figure 2).

**Figure 3.**
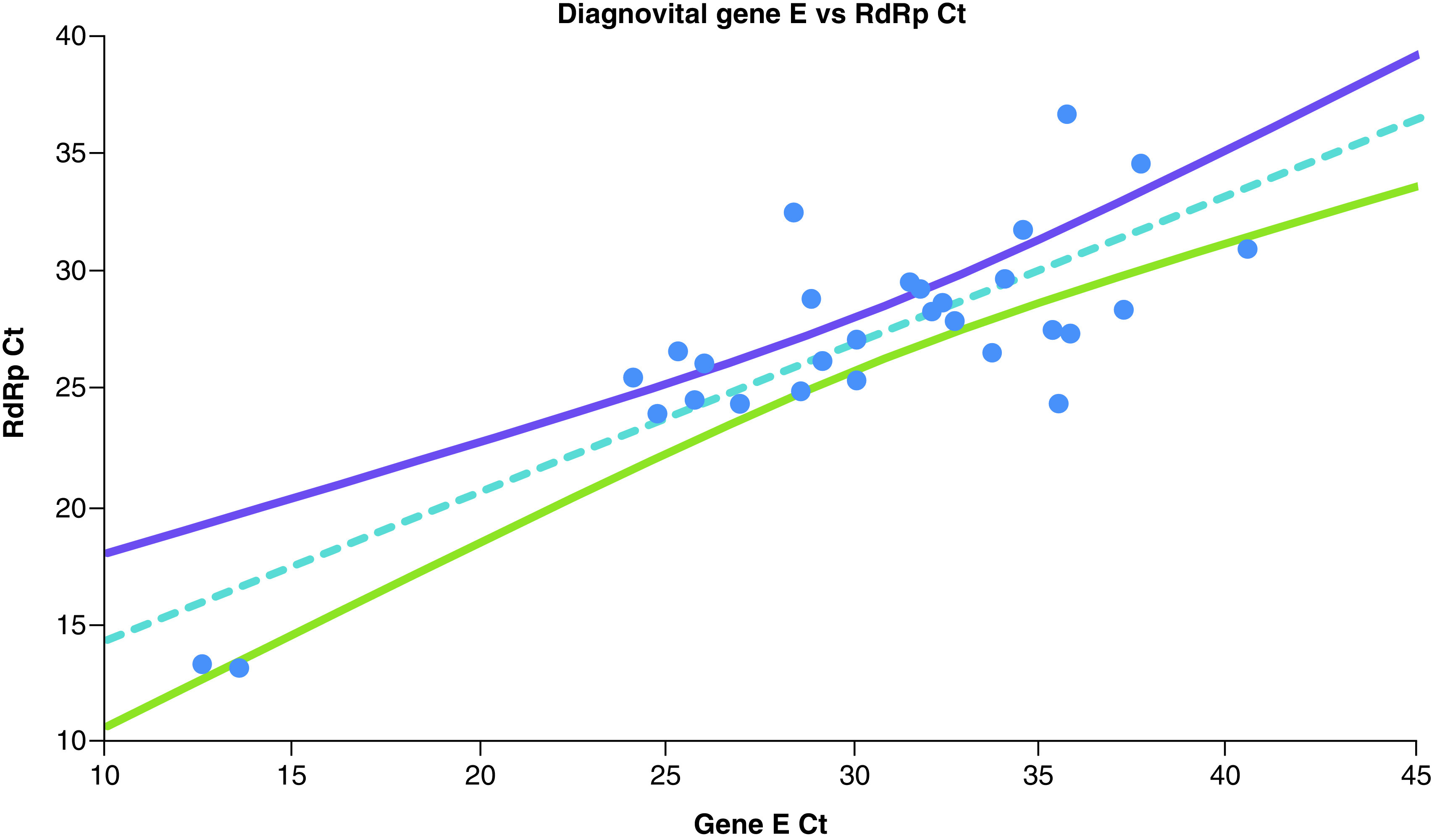
The Deming regression of Ct values obtained for *RdRp* gene and gene *E* for Diagnovital^®^ and 95% CI for the regression line. Any data pair with any negative results are omitted.

The Deming regression of Ct values obtained for *RdRp* gene and gene *E* for Diagnovital and 95% CI for the regression line shown in Figure 3. The two biomarkers used by Diagnovital, gene *E* and *RdRp* gene were also showed some degree of correlation. This correlation was found statistically significant (p < 0.01) with a Pearson correlation of 0.82 (Figure 3).

**Figure 4.**
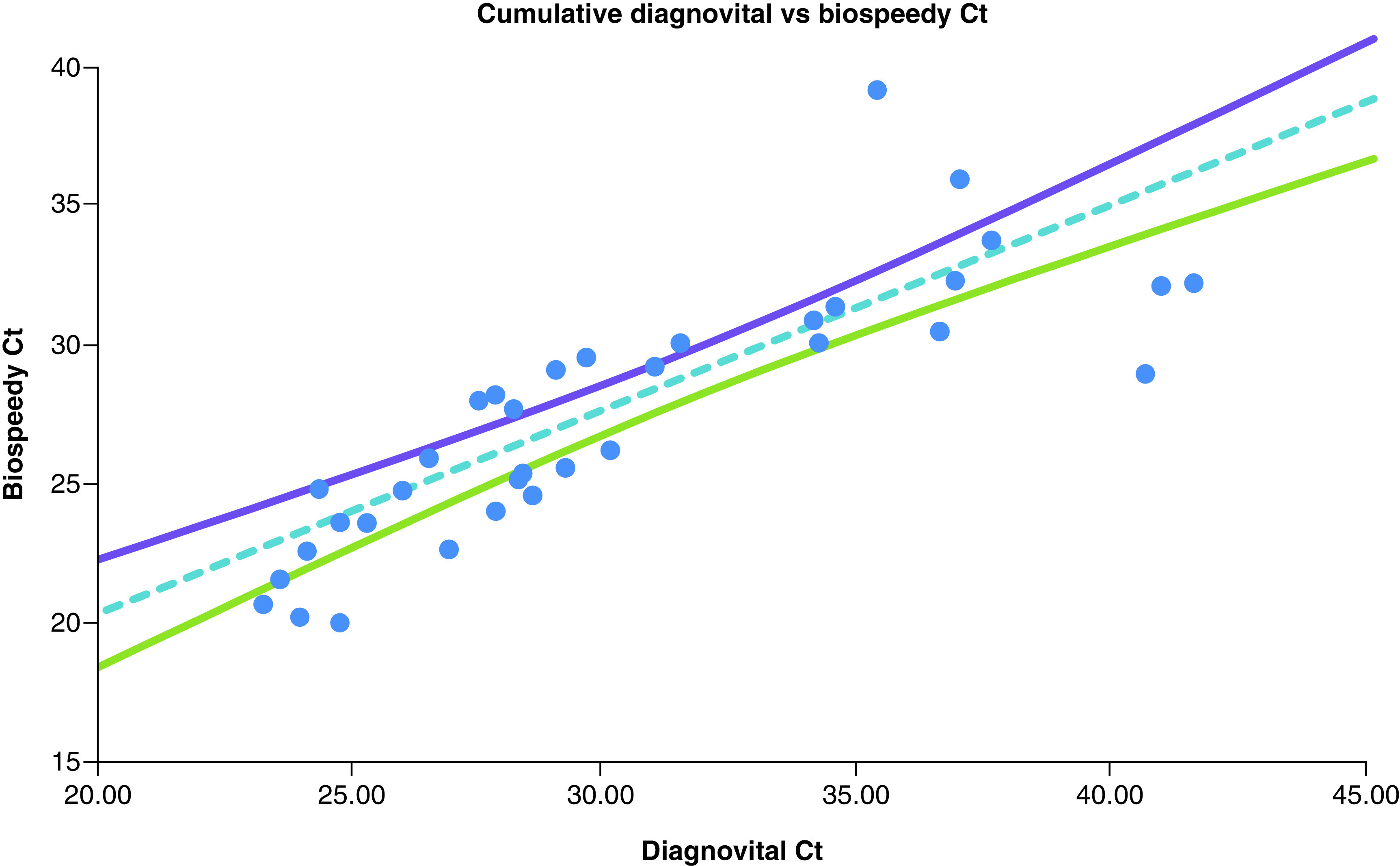
The Deming regression of the cumulative Ct values of Diagnovital^®^ and *RdRp* Ct values of Biospeedy^®^ and 95% CI for the regression line. The cumulative Ct values were calculated by the sum of arbitrary copy numbers of gene *E* and *RdRp* genes obtained through 2-Ct. The arbitrary copy numbers for negatives were assumed as zero.

Figure 4 shows the Deming regression of the cumulative Ct values of Diagnovital and *RdRp* Ct values of Bio-speedy and 95% CI for the regression line. The cumulative Ct values were calculated by the sum of arbitrary copy numbers of gene *E* and *RdRp* genes obtained through 2^-Ct^. The cumulative scores, when compared with Bio-speedy results, showed statistically significant (p < 0.01) correlation with a Pearson coefficient of 0.83. This value being higher than 0.73 coefficient obtained through comparison of *RdRps* of the two kits only, showed that inclusion of a secondary biomarker by Diagnovital improved the correlation of different kits.

**Figure 5.**
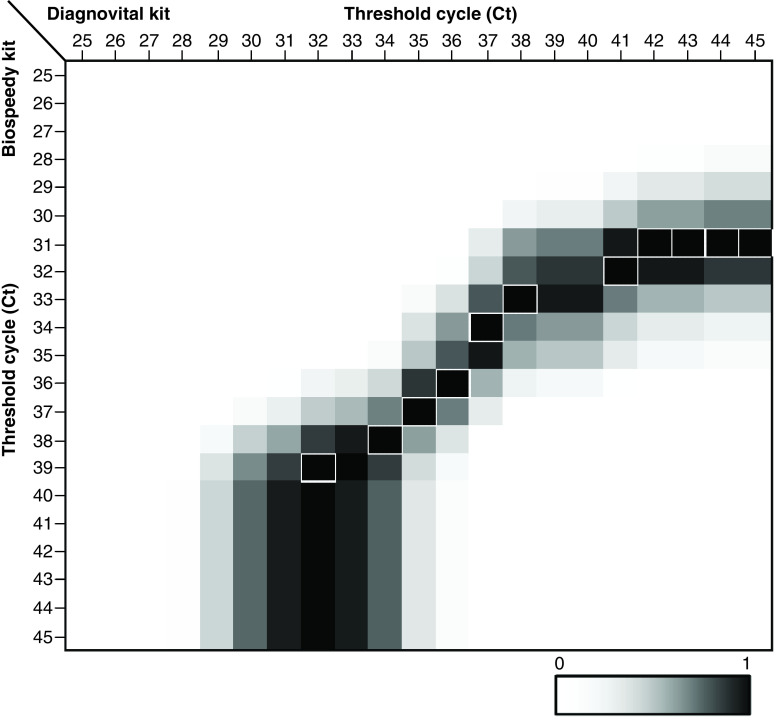
Heat map for chi-square scores obtained for a range of thresholds for Biospeedy^®^ and Diagnovital^®^ kits. Black and white squares indicate chi-square score of 1 and 0, respectively. The black squares with white borders indicate Ct threshold combinations where chi square scores are above 0.88.

In Figure 5 the analyzed heat map for chi-square scores obtained for a range of thresholds, from 25 to 45, encapsulating the manufacturer defined thresholds, for Bio-speedy and Diagnovital kits are shown. Black and white squares indicate chi-square score of 1 and 0, respectively. According to chi-square heat map, while the two kits showed high independence for manufacturers choice of Ct, there was a range of Ct combinations for the two where the two kits showed a high correlation. When the Ct of 40 for Bio-speedy and 35 for Diagnovital was applied as instructed by the manufacturers, the chi-square score of 0.18 was obtained, indicating a high independence between the kits for manufacturer defined Cts. On the other hand, the maximum dependence for the two kits could be established if the Ct were modified to any of the 38 and 32, 38 and 34, 37 and 35, 34 and 37 combinations for Bio-speedy and Diagnovital, respectively. While dependence could increase at even higher Ct values, it is important to note that high dependence is not an indication of high sensitivity or specificity.

## Discussion

On January 26, the first batch of four registration certificates of Novel Coronavirus PCR kit was issued by the State FDA for the emergency, without a series of clinical trials [17]. Then, new PCR kits began to be produced by different laboratories and institutes. Many studies on the obtained kits have also been carried out and are still underway [18]. Nowadays, Turkey is among the countries producing SARS-CoV-2 diagnostic PCR kits. In this study, we investigated the compatibility between the two different SARS-CoV-2 PCR kits produced in Turkey during the COVID-19 pandemic. We used two separate SARS-CoV-2 RT-PCR kits similar to each other, including the procedure, analytic sensitivity, storage of the kits, studied samples and other features shown in Table 1. The differences were the *E* gene analysis in addition to *RdRp* gene in the Diagnovital PCR kit and isolation methods. Our results indicate, while the majority of samples showed correlation regarding the diagnostic decision between the two kits, there were conflicts due to viral load close to the thresholds defined by the manufacturers. For that reason, in the absence of international quality controls, it is our conclusion that accurate diagnosis was possible with a high positive cumulative correlation of kits.

In COVID-19 infection, viral RNA in the nasopharyngeal swab, measured by RT-PCR, can be detected in most individuals a week before the onset of symptoms and peaked in the first week of symptom onset. Ct is the number of replication cycles required to produce a fluorescent signal with lower Ct values indicating higher viral RNA loads. Those with PCR positive will have a Ct value of less than 40 as defined by the manufacturer [18,19]. It was interesting that varying degrees of differences in Cts were measured using different kits for the same patient samples. Assuming starting quantities were the same, they had different yield quality. Especially, Diagnovital PCR kit showed in a narrow Ct range and conveniently sharper amplification curves than Bio-speedy PCR kit (Figure 1). Nevertheless, the strong correlation of the two kit results suggested that two different RNA targeting gene assays were appropriate as suggested by WHO in the diagnosis of COVID-19 disease [20]. In general, it is difficult to understand the analytical sensitivity differences of different molecular tests due to natural variations in sample processing and reference materials used for validation in different laboratories. However, the study comparing three different molecule tests by Uhteg *et al.* showed that similar results were found; the PCR kit with two different genes of the SARS-CoV-2 had a higher yield than the other two kits performing one gene analysis [21].

*RdRp* assays had been implemented in 30 laboratories in Europe [20]. The WHO recommended the detection of at least two different targets on the SARS-CoV-2 genome [22]. It is also recommended that *E* gene assay as a primary screening tool is followed by the *RdRp* gene assay as confirmatory tests [23,24]. Our data suggested that both of the PCR methods yielded comparable results (a Pearson coefficient of 0.83) for both negative and positive clinical specimens (Figure 4). However, we did find a notable difference in comparison of *RdRps* of the two kits with a correlation of 0.73 (Figure 2). It was important to note that since the isolation methods and possibly primers were different, we did not expect to observe a slope of 1. Indeed, Bio-speedy showed higher variation when it comes to Cts, yet the correlation was statistically significant (p < 0.01). While the two kits reported similar results for the *RdRp* gene, there was a considerable difference as well. The addition of a secondary biomarker for Diagnovital improved the correlation of different kits and increased the correlation by raising it to 0.83 (Figure 4). While neither necessarily represent the true values, the increased correlation was an indication of improved diagnostic power. On the other hand, as shown in Figure 3, both biomarkers were somewhat correlated with the 95% CI for the Deming regression line of the Ct values obtained for the Diagnovital for the *RdRp* gene and the *E* gene. This result indicated that the two biomarkers reported relatively close results adding to the cumulative detection power.

As seen in Diagnovital, the inclusion of secondary biomarkers, although minimal, do add to the correlation and possibly to the diagnostic power. We have to remember that during a pandemic, time and cost are both important factors affecting the manufacturer’s choices, however, any misdiagnosed patient, as in, false negative, could increase the burden of the pandemic on the health system dramatically and this should be taken into consideration by the associated governmental bodies when defining kit standards.

As in any qualitative assay, choosing a threshold level is essential to finalize the diagnosis and classify the patient. Most diagnostic tools refer to any Ct value above a specific threshold, as too low for detection and refrain from a conclusive classification. In the case of a pandemic event, the doctors, however, do not have the luxury or resources. Unfortunately, without true positives or true negatives, we do not have the means to perform receiver operating characteristic analyses in order to determine the right thresholds to reach an optimum diagnostic power. Despite this, we had the means to compare the two diagnostic tools and how they were correlated with each other. Using Deming, while it was possible to correlate the Cts; however, the thresholds for diagnosis could not be correlated because diagnosis was categorical rather than continuous data, as in Cts. Instead, we had the means of correlation of categorical data using the chi-square score as a specific threshold combination. However, depending on the choice of thresholds, this score was prone to change. Performing a grid search, we scanned a range of thresholds for both kits, producing a heatmap of chi-square scores (Figure 5). It is important to note that the heatmap did not provide information on accuracy, but rather, correlation of the kits. According to the chi-squares heatmap, the range of high correlation thresholds is along the diagonal of Ct ranges. Surprisingly, the correlation dramatically dropped outside of this diagonal, including the threshold combination determined by the kit manufacturers (35 for Diagnovital and 40 for Bio-Speddy). This case indicated that while the correlation for Cts exists, the choice of threshold might strongly influence the diagnosis. To obtain a higher accuracy, a combinatory study was advisable to determine optimum thresholds using multiple kits for even a few hundred cases.

The RT-PCR tests today are so new that it is unclear how reliable they are. Local laboratories are doing their best to validate tests on their own. They also aim to reach larger masses by performing more tests and thus preventing the spread of the disease by detecting patients as soon as possible. In our country Scientific and Technological Research Council of Turkey had also accepted all projects about PCR kits. This was conducted because the country needed these kits to help with quality assurance internally. A viewpoint published in JAMA earlier this month synthesized known data on the accuracy of different tests at different points in the disease process target genes for verification test [25]. The confirmatory test result is considered positive only when both confirmatory genes are detected. If a gene is not detected, the result cannot be interpreted positively [16].

In conclusion, it should be noted once again that the international external quality controls for the diagnosis of SARS-CoV-2 by PCR techniques used have not been established as of the writing of this manuscript. Our results suggest, in the light of clinic symptoms of COVID-19 using different PCR kits that target different genes concurrently during the pandemic may provide a more accurate diagnosis.

## Conclusion

Diagnovital PCR kit showed a narrower cycle range of amplification and conveniently sharper amplification curves than Bio-speedy PCR kit. This case suggested that two different RNA target gene assays were more appropriate as suggested by WHO in the diagnosis of COVID-19 disease. Both of the PCR methods yielded comparable results for both negative and positive clinical specimens. However, we did find a notable difference comparison of *RdRps* of the two kits. Both biomarkers were somewhat correlated with the 95% CI for the Deming regression line of the Ct values obtained for the Diagnovital for the *RdRp* gene and the *E* gene. This result indicated that the two biomarkers reported relatively close results adding to the cumulative detection power. While the correlation for Cts exists, the choice of threshold might strongly influence the diagnosis. To obtain a higher accuracy, a combinatory study was advisable to determine optimum thresholds using multiple kits for even a few hundred cases. Our results suggest, in an unknown sample and in light of clinic symptoms of COVID -19, using different PCR kits that possibly target different genes may provide a more accurate diagnosis.

**Summary points**

**Background**

- In the diagnosis of severe acute respiratory syndrome coronavirus 2, reverse transcriptase-PCR (RT-PCR) technique is often used. Various RNA gene targets are used.
- Until now, international external quality controls of the kits used have not been established.
- We evaluated the compatibility of severe acute respiratory syndrome coronavirus 2 RT-PCR kits containing different gene targets during the coronavirus disease 2019.

**Materials & methods**

- Oro/nasopharyngeal swab samples were isolated symptomatic/asymptomatic patients tested by Bio-Speddy^®^ (*RdRp* gene) and Diagnovital^®^ (*RdRp + E* genes) assays simultaneously.

**Results**

- Diagnovital PCR kit showed in a narrower cycle range and conveniently sharper amplification curves than Biospeedy PCR kit.
- The *RdRp* of the two kits had a correlation of 0.73 (Pearson coefficient), while the correlation increased to 0.83 when a secondary biomarker (gene *E*) was added to the Diagnovital PCR kit.

**Conclusion**

- Our results suggest, in an unknown sample and in light of clinical symptoms of COVID-19, using different PCR kits that possibly target different genes may provide a more accurate diagnosis.
